# Competitive Anxiety and Mood States in High-Performance Cuban Student Athletes

**DOI:** 10.11621/pir.2024.0304

**Published:** 2024-09-15

**Authors:** Jesús Ríos-Garit, Marta Cañizares-Hernández, Mario Reyes-Bossio, Yanet Pérez-Surita, Raul Touset-Riverí

**Affiliations:** a *Central University “Marta Abreu” of Las Villas, Santa Clara, Cuba*; b *University of Sciences of Physical Culture and Sport “Manuel Fajardo”, Havana, Cuba*; c *Peruvian University of Applied Sciences, Lima, Peru*

**Keywords:** competitive anxiety, dual career, gender, mood states, sport type, high performance

## Abstract

**Background.:**

The study of competitive anxiety and its relationship with mood states in high-performance athletes is relevant for predicting performance and enabling timely interventions to ensure successful outcomes in competitions. Due to the complex psychological demands arising from dual careers, the study of competitive anxiety and mood states contributes valuable insights into the emotional well-being of these student athletes.

**Objective.:**

To examine and describe competitive anxiety and mood states in a sample of high-performance Cuban university athletes across different sports and genders.

**Design.:**

A descriptive, correlational, and cross-sectional study was conducted involving 46 Cuban student athletes from national teams across 16 sports and included both male and female athletes, with an average chronological age of 20.70 years and 6.98 years of experience in a high-performance sport. The *Competitive Sport Anxiety Inventory* and *Brunel Mood Scale* were administered in their Spanish versions. The data were examined using descriptive statistics analysis including the Mann-Whitney U test, and Spearman’s correlation coefficient.

**Results.:**

Although no statistically significant differences were found in competitive anxiety and mood states in relation to gender and type of sport, female athletes tend to present higher mean scores in competitive anxiety and negative mood states. Additionally, female athletes in team sports experienced slightly more intense emotions. Anxiety shows positive correlations with tension, depression, and vigour, suggesting its influence on certain mood states.

**Conclusion.:**

The results indicate that, in general, the intensity of certain moods in Cuban university student athletes is influenced by levels of competitive anxiety. An increase in anxiety during competition can lead to heightened tension, depression, and vigour, with no significant differences observed between female and male athletes or based on the type of sport practiced.

## Introduction

The psychological determinism of sports performance has been articulated in various theoretical models emphasize the mental skills athletes need to manage the stress of competitive activity and training (Loehr, 1986; Mahoney, 1987; Smith et al., 1995; Vealey, 2007). According to Ríos-Garit (2021), an athlete’s success largely depends on the configuration of the cognitive and affective aspects of their personality, as this determines how they manage adaptive processes in potentially stressful situations. Effective coping in these contexts can significantly influence the distinction between success and failure in high-level sport.

In addition to psychological skills, researchers have also analysed the role of negative emotions in predicting sports performance, yielding important findings that underscore the significance of viewing the athlete as a biopsychosocial entity (Terry & Parsons-Smith, 2021). This suggests that in addition to physical, technical, and tactical aspects (Cantón & Checa, 2012; Domínguez-González et al., 2024), an athlete’s performance is also influenced by the management and understanding of emotions, particularly anxiety levels, which have been found to impact competitive performance and the effectiveness of training (Cañizares, 2004).

Competitive anxiety is a common phenomenon that affects sports performance in various ways (Martens et al., 1990). If not properly managed during competition, anxiety can cause a drop in performance and lead to even among athletes with high levels of physical and technical preparation (Jaramillo et al., 2020; Menéndez-Fierros & Becerra Hernández, 2020; Triqueros et al., 2020). On the other hand, achieving and maintaining an optimal mood is considered an important part of mental preparation for achieving excellence in sport performance (Arruza et al., 2011; Feria-Madueño et al., 2023; López-Torres, et al., 2007).

Peñaloza-Gómez et al. (2016) researched the influence of competitive anxiety on the mood states of athletes in a broad and heterogeneous sample of female and male athletes from several disciplines. The results revealed significant differences in anxiety and mood states based on sex. Additionally, both cognitive and somatic dimensions of competitive anxiety were found to predict negative mood states, while self-confidence positively predicted vigour and negatively predicted confusion. Based on these findings, the authors determined that anxiety interacts with various mood states that negatively impact the performance of athletes.

The relationship between emotional states and physical activity has been studied for several decades by comparing mood profiles of athletes and exercise practitioners with non-practitioners. These studies have consistently revealed improvements in mood after performing physical exercises (Álvarez-Muñoz et al., 2023; Barrios-Duarte, 2006; Pereira-Gaia et al., 2021). Regarding high-performance university athletes, some studies indicate that dual careers offer benefits for personal development (Harrison et al., 2020; Reyes-Hernández et al., 2021; Reyes-Bossio et al., 2023).

Despite these findings, high-performance university athletes face complex psychological and social demands due to the simultaneous engagement in sports practice and academic study as their primary activities (Massó et al., 2022). The implications of these demands have diverse effects for the athletes, significantly impacting their mental health and poses risks to their emotional well-being (Reyes-Bossio, 2020; Reyes-Bossio et al., 2023; Schinke et al., 2017). This became particularly evident during the recent COVID-19 pandemic, highlighting the importance of adherence to sport psychology (Barbosa-Granados, Arenas-Granada et al., 2022).

Consequently, research in university sport is becoming increasingly frequent, revealing findings that underscore the need to incorporate studies focused on the performance and psychological characteristics of these athletes. Several studies have highlighted the complexity of the simultaneous influence of academic study and sport on the performance of individuals in both activities (Capranica et al., 2021; Conde et al., 2021; Stambulova & Wylleman, 2019; Torregrossa et al., 2020).

Although Cuba provides support to university athletes through ongoing monitoring of their sports and academic lives driven by governmental interest (Massó et al., 2022), scientific studies on the emotional characteristics of these individuals engaged in dual career from an early age until their transition to university life have been insufficient. Therefore, this research aims to characterize competitive anxiety and mood states in a sample of high-performance Cuban university athletes across different sports and genders.

## Methods

A descriptive, correlational, and cross-sectional study was conducted to characterize the competitive anxiety and the mood states in high-performance university athletes from the University of Sciences of Physical Culture and Sport “Manuel Fajardo”.

### Participants

46 university student-athletes pursuing a degree in physical culture participated, representing national teams from 16 different sports. A heterogeneous sample was formed in terms of type of sports (25 team sports and 21 individual sports), but equivalent in terms of gender (23 female and 23 male). The athletes had an average chronological age of 20.70 years and an average of 6.98 years of experience in high performance sport.

An intentional sampling was carried out based on the following criteria:

*Inclusion:* All the athletes were in the training preparation phase.

*Exclusion:* Athletes who do provide their consent to participate in the study.

*Exit:* Athletes who do not complete all the instruments for psychological evaluation.

*Table 1* describes the specific sports and their classification as either a team or individual sport.

**Table 1:** Description of the sample according to type of sport

Nº	Sports	Type	Frequency	Gender		Total
				Female	Male	
1	Soccer	Team sport	9	2	7	25
2	Basketball	Team sport	6	4	2	
3	Volleyball	Team sport	5	2	3	
4	Baseball	Team sport	2	0	2	
5	Beach Volleyball	Team sport	2	1	1	
6	Hockey	Team sport	1	1	0	
7	Gymnasia	Individual sport	4	4	0	21
8	Olympic wrestling	Individual sport	4	0	4	
9	Karate	Individual sport	3	0	3	
10	Sport’s shot	Individual sport	2	2	0	
11	Weightlifting	Individual sport	2	2	0	
12	Chess	Individual sport	2	1	1	
13	Taekwondo	Individual sport	1	1	0	
14	Swordplay	Individual sport	1	1	0	
15	Athletics	Individual sport	1	1	0	
16	Judo	Individual sport	1	1	0	
	Total			23	23	46

### Materials

The Spanish version of the *Competitive Sport Anxiety Inventory* (Martens et al., 1990; Márquez, 1992) was used to evaluate competitive anxiety. The instrument comprises 27 items distributed across three subscales that measure cognitive, somatic anxiety and self-confidence, with four Likert-type response options: (1 = Not at all; 2 = A little; 3 = Moderately; 4 = A lot). The total score across the three scales was considered, resulting in a Cronbach’s Alpha coefficient of .95.

The Spanish version *of the Brunel Mood Scale* (Mcnair et al., 1971; Cañadas et al., 2017) was applied to provide an evaluation of mood states in adolescent and adult populations. It consists of 24 items that describe simple mood states. Responses are recorded using a 5-point Likert-type scale: (1 = Not at all; 2 = A little; 3 = Moderately; 4 = Quite a bit; and 5 = Extremely). The instrument has six subscales, with Cronbach’s alpha coefficient of .85 for Tension, .71 for Depression, .75 for Anger, .78 for Vigour, .67 for Fatigue and .80 for Confusion.

### Procedure

After obtaining informed consent from the participants, the instruments were administered in the morning in printed format in the classrooms of the Manuel Fajardo University of Physical Culture and Sports Sciences, under optimal conditions for completion, following a careful explanation of the instruments’ characteristics and objectives. Two consecutive days were taken for the application of the instruments over two consecutive days (one each day).

### Data analysis

The data were analysed using descriptive statistics such as the mean, standard deviation, skewness, and kurtosis. The Kolmogorov-Smirnov test was applied and when a lack of normality was determined, the Mann-Whitney U test was applied to compare the psychological variables between athletes according to gender (female or male) and type of sport (individual or team). Spearman’s correlation coefficient was used to determine bivariate correlations between competitive anxiety, mood states, chronological age, and high-performance sport experience. The statistical software SPSS V. 25.0 for Windows was used for data analysis.

## Results

In *Table 2* shows that depression, anger and vigour have higher mean values than the other mood states. On the other hand, competitive anxiety indicates greater dispersion than the other variables but follows a normal distribution. Most variables do not exhibit normality.

**Table 2:** Description of the variables under study and normal distribution test

Variables	Mean	Standard deviation	Skewness	Kurtosis	Z	p
Chronological age	20.70	2.56	3.422	16.153	.253	.000
High-performance sport experience	6.98	4.45	.825	–.257	.196	.000
Competitive Anxiety	39.54	9.84	.422	.475	.102	.200
Tension	6.61	3.07	1.564	2.281	.198	.000
Depression	9.17	3.38	.067	–.660	.118	.113
Anger	8.13	3.10	.932	1.074	.169	.002
Vigour	7.00	3.36	1.217	.940	.186	.000
Fatigue	6.65	3.30	1.310	1.047	.235	.000
Confusion	6.20	3.36	1.906	3.066	.257	.000

*Note. Z= Kolmogorov-Smirnov.*

*Table 3* it is observed that no variable establishes significant difference based on gender, however, in general sense, girls have greater competitive anxiety and negative mood states, except fatigue.

**Table 3:** Mood states and competitive anxiety between female and male athletes

Variables		N	Average range	Mann-Whitney U	*p*
Competitive Anxiety	Female	23	23.70	260.00	.921
	Male	23	23.30		
Tension	Female	23	25.83	211.00	.229
	Male	23	21.17		
Depression	Female	23	23.89	255.50	.842
	Male	23	23.11		
Anger	Female	23	23.83	257.00	.868
	Male	23	23.17		
Vigour	Female	23	24.15	249.50	.735
	Male	23	22.85		
Fatigue	Female	23	22.85	249.50	.732
	Male	23	24.15		
Confusion	Female	23	26.17	203.00	.151
	Male	23	20.83		

*Table 4* shows that, although no significant differences were found, athletes in team sports have higher scores in mood states and competitive anxiety.

**Table 4:** Mood states and competitive anxiety between team and individual sports

Variables		N	Average range	Mann-Whitney U	*p*
Competitive Anxiety	Team Sports	25	23.62	259.50	.947
	Individual Sports	21	23.36		
Tension	Team Sports	25	25.38	215.50	.289
	Individual Sports	21	21.26		
Depression	Team Sports	25	24.24	244.00	.681
	Individual Sports	21	22.62		
Anger	Team Sports	25	26.22	194.50	.130
	Individual Sports	21	20.26		
Vigour	Team Sports	25	24.90	227.50	.429
	Individual Sports	21	21.83		
Fatigue	Team Sports	25	25.00	225.00	.390
	Individual Sports	21	21.71		
Confusion	Team Sports	25	23.94	251.50	.797
	Individual Sports	21	22.98		

*Table 5* shows significant and positive correlations between mood states. In contrast, competitive anxiety demonstrates meaningful positive relationships only with tension, depression, and vigour.

**Table 5:** Correlation between chronological age, experience in high-performance sports, mood states and competitive anxiety

Variables		1	2	3	4	5	6	7	8
1.	Chronological age								
2.	High-performance sport experience	–.080							
3.	Tension	.023	.053						
4.	Depression	–.155	.057	.581**					
5.	Anger	–.011	.050	.688**	.803**				
6.	Vigour	.054	.052	.776**	.627**	.694**			
7.	Fatigue	.106	.082	.712**	.681**	.821**	.810**		
8.	Confusion	.012	–.075	.704**	.469**	.691**	.697**	.738**	
9.	Competitive Anxiety	–.170	.244	.299*	.324*	.289	.295*	.288	.217

Note. *p<.05; **p<.01 (two-tailed)

## Discussion

The study found that student athletes had a predominantly negative mood and competitive anxiety which did not differ statistically between female and male athletes. No significant differences were found between team and individual sports athletes. Furthermore, mood states were found to be positively and significantly related to each other, denoting systemic interdependence among mood states, while competitive anxiety was found to influence levels of tension, depression and vigour. Specifically, a higher level of anxiety in competition may be accompanied by an increase in these mood states, which may condition sport performance.

The mood states with the highest scores were depression, anger, and vigour in that order. This indicates a predominance of negative emotions at the time of the study, which contrasts with the findings of Oliveira et al. (2020) in a sample of basketball athletes, who exhibited a predominantly positive mood, as vigour obtained the highest scores at the beginning and end of the season.

No significant differences were found in mood states between female and male athletes, consistent with the findings of Castro-Sánchez et al. (2018), but differing from the results of Cañadas et al. (2017), who determined that female athletes exhibited greater anger, confusion, depression, and fatigue, but less vigour than male athletes. Similarly, a study by Peñaloza-Gómez et al. (2016) showed that female athletes scored lower on vigour and higher on confusion. Other research found that female athletes tend to present more negative emotional states (Balaguer et al., 1993; McDowell et al., 2016).

Although no statistically significant differences were found, it is striking that female athletes had higher mean scores in tension, depression, anger, vigour, and confusion, while male athletes scored higher in fatigue. Therefore, it is understood that female athletes tend to experience more intense emotions than male athletes, including negative and positive emotions. These results differ from the study by Romero-Martín et al. (2017 which found that men expressed more intense emotions than women; however, they coincide with the majority of previous research across both athlete and non-athlete populations (Cañadas et al., 2017; Peñaloza-Gómez et al., 2016; Terry et al., 2021).

Competitive anxiety also showed higher average scores in female athletes which is consistent with several studies that indicate a trend towards higher anxiety rates in women (Castro-Sánchez et al., 2020; Menéndez-Fierros & Becerra-Hernández, 2020; Peñaloza-Gómez et al., 2016). The divergent and concurrent results from both investigations regarding the comparison of mood states between female and male athletes implies the need for further research. However, there is greater agreement among several studies on competitive anxiety between women and men, indicating that female athletes are more susceptible to experiencing high levels of anxiety in competition.

Conversely, no significant differences were found in the levels of competitive anxiety and mood states in the athletes across the various types of sport practiced. However, higher average scores were observed for team sports. This suggests that, despite emotional affect is relatively consistent across types of sports, team sports elicit higher levels of stress or emotional burdens on athletes.

These observations are consistent with a study by Castro-Sánchez et al. (2018) which determined that team sports could require a greater degree of emotional skills due to the simultaneous interactions between teammates and opponents during games. These differentiating observations between team and individual sports could have influenced the results of this research, since, according to Terry (1997), the effects upon moods appear to be mediated by various factors including the type of sport practiced.

The correlational analysis showed that age and experience in high-performance sports are not associated with competitive anxiety or mood states, which contrasts with the findings of several authors who have determined that older and more experienced athletes better control the emotional effects of sporting situations. (Hernández et al., 2008; Peñaloza-Gómez et al., 2016). Based on the above, it was expected that athletes with more experience would present more positive moods and less anxiety in the competition. The absence of a relationship between sports experience and psychological variables associated with performance was also found by Ríos-Garit et al. (2023) in a study on young athletes from different team and individual sports. In both cases, the average experience of the athletes was not notably high, which could have influenced the findings obtained in both investigations.

On the other hand, mood states have strong relationships of positive interdependence, while anxiety is only positively related to tension, depression, and vigour. This suggests that the increased anxiety during competition is typically accompanied by heightened mood intensity, exacerbating the negative emotional experiences of athletes. Feria-Madueño et al. (2023) also observed the relationship between tension and anxiety in youth athletes during training sessions at the Cuban national athletics preselection events.

The findings suggest that the athletes have emotional experiences that can negatively or positively influence performance during the upcoming competition due to the relationships established between competitive anxiety and two of the three most intense mood states: depression and vigour. The influence of competitive anxiety on the moods of athletes has been illustrated by Peñaloza-Gómez et al. (2016). By analysing 255 athletes from 26 different sports, these authors determined that cognitive and somatic anxiety predicted negative mood states such as anger, depression, fatigue, tension, and confusion. They also found that somatic anxiety and self-confidence appear to be positively predicated on vigour. Furthermore, self-confidence also predicted negative behaviour associated with confusion.

The results of this research confirm the importance of athletes and technical staff being aware of competitive anxiety and emotional states in sport. Providing workshops, talks or educational material can help increase awareness of this topic (Barbosa-Granados, Castañeda-Lozano et al., 2022) and incorporating an approach that addresses economic, financial, educational, competitive, and social aspects is especially important (Håkansson et al., 2020).

## Conclusion

Although no notable differences were found between female and male athletes, a marked trend in presenting negative mood states and greater competitive anxiety in female athletes was observed. Likewise, in team sports, slightly more intense negative and positive moods are evident than observed for individual athletes. The results obtained indicate that overall, the intensity of negative moods in these Cuban university athletes depended on their level of competitive anxiety, given that an increase in competitive anxiety can lead to greater tension, depression, and vigour.

## Limitations

The cross-sectional and correlational design of the research limits the analytical scope of the results obtained, as it does not allow for the explanation or prediction of the influence of competitive anxiety on the moods of high-performance Cuban university athletes. The findings have limited generalizability due to the size of the sample and its low representativeness of the population.
